# Adolescent Major Depressive Disorder: Neuroimaging Evidence of Sex Difference during an Affective Go/No-Go Task

**DOI:** 10.3389/fpsyt.2017.00119

**Published:** 2017-07-11

**Authors:** Jie-Yu Chuang, Cindy C. Hagan, Graham K. Murray, Julia M. E. Graham, Cinly Ooi, Roger Tait, Rosemary J. Holt, Rebecca Elliott, Adrienne O. van Nieuwenhuizen, Edward T. Bullmore, Belinda R. Lennox, Barbara J. Sahakian, Ian M. Goodyer, John Suckling

**Affiliations:** ^1^Department of Psychiatry, University of Cambridge, Cambridge, United Kingdom; ^2^California Institute of Technology, Pasadena, CA, United States; ^3^Department of Psychology, Columbia University, New York, NY, United States; ^4^Cambridgeshire and Peterborough NHS Foundation Trust, Cambridge, United Kingdom; ^5^Behavioural and Clinical Neuroscience Institute, Cambridge, United Kingdom; ^6^Institute of Brain, Behaviour and Mental Health, University of Manchester, Manchester, United Kingdom; ^7^University of Cambridge, Cambridge, United Kingdom; ^8^Department of Psychiatry, University of Oxford, Oxford, United Kingdom

**Keywords:** adolescent major depressive disorder, affective go/no-go task, cerebellum, supramarginal gyrus, sex difference

## Abstract

Compared to female major depressive disorder (MDD), male MDD often receives less attention. However, research is warranted since there are significant sex differences in the clinical presentation of MDD and a higher rate of suicide in depressed men. To the best of our knowledge, this is the first functional magnetic resonance imaging (fMRI) study with a large sample addressing putative sex differences in MDD during adolescence, a period when one of the most robust findings in psychiatric epidemiology emerges; that females are twice as likely to suffer from MDD than males. Twenty-four depressed and 10 healthy male adolescents, together with 82 depressed and 24 healthy female adolescents, aged 11–18 years, undertook an affective go/no-go task during fMRI acquisition. In response to sad relative to neutral distractors, significant sex differences (in the supramarginal gyrus) and group-by-sex interactions (in the supramarginal gyrus and the posterior cingulate cortex) were found. Furthermore, in contrast to the healthy male adolescents, depressed male adolescents showed decreased activation in the cerebellum with a significant group-by-age interaction in connectivity. Future research may consider altered developmental trajectories and the possible implications of sex-specific treatment and prevention strategies for MDD.

## Introduction

### Sex Differences in Major Depressive Disorder (MDD)

The incidence of MDD for girls rises from age 11 to 13 years until by age 15 one of the most robust findings in psychiatric epidemiology emerges; that females are twice as likely to suffer from MDD than males (although this predominance might disappear after age 55 years) (1). Possible explanations for sex differences in the incidence of adolescent MDD include: girls having more negative thinking styles, ruminating on interpersonal and body image events, having greater hormone fluctuations, reporting more negative events and sexual abuse, a higher vulnerability to inflammation (2), and estrogen-induced stress response enhancement of the prefrontal cortex (3). The genetic risk for MDD also differs strikingly between sexes with more heritability in women (4). In terms of other risk factors, social stress is closely related to female MDD, whereas low self-esteem is more likely to be related to male MDD (5). Furthermore, looking after small children is associated with greater risk of MDD in women (6) whereas men are more sensitive to the depressogenic effects of divorce, separation, and occupational problems (7). Consequently, research into MDD occurring at this early stage of adolescence avoids these complicating social factors.

Despite strong evidence suggesting poor outcomes, male MDD is underrepresented in the extant literature. Men are more liable to persistent depression whereas women tend to suffer from a more episodic disorder (8). Depressed men are also more likely to commit suicide (9) and abuse substances (10) than depressed women. Suicidal attempts and substance abuse have been related to poor cognitive control (11, 12). Significant sex differences have been found in the cognitive control of emotion (13). Taken together, exploration of cognitive control, and especially of emotional cognitive control, might facilitate an understanding of sex differences in MDD.

### Cognitive Control in MDD

Cognitive control can be defined as neurocognitive processes important for achieving goals, particularly when adaptive adjustment is needed in response to changing environmental demands (14). The possible causal relationship between impaired cognitive control and MDD was proposed as early as Beck’s Cognitive Theory of Depression in 1979 (15). Indeed, impaired cognitive control has been associated with the vulnerability, onset, and maintenance of MDD (16). It has also been suggested that persisting cognitive deficits lead to poor psychosocial functioning and hence poor quality of life in remitted depressed patients.

This study used functional magnetic resonance imaging (fMRI) to explore cognitive control among emotional distractions during a widely used affective go/no-go (AGNG) task. In this task, participants responded by button press to target (“go”) stimuli that were emotionally valent (e.g., sad) while inhibiting their response to distractor (“no-go”) stimuli of a different valence (e.g., happy). The cingulate, inferior frontal gyrus, and insula cortex are most frequently activated during the inhibition of emotional distractors in the AGNG task (17–25). These three regions are also commonly reported in non-affective go/no-go studies when comparing no-go and go events (26–30) with additional regions such as the middle temporal gyrus, cerebellum and the striatum frequently reported in adolescents (24, 31–36).

Compared to healthy adults, depressed adults show increased activation in the right lateral orbitofrontal cortex and bilateral anterior temporal cortex in response to sad versus neutral distractors (37) which indicates an activation bias toward negative information. Nevertheless, our recently published study using the same task found aberrant brain activation in the orbitofrontal cortex of female adolescents with MDD in response to the happy versus neutral distractors (38). Consequently, it is not clear whether happy, sad, or both distractors elicit aberrant brain responses in adolescents with MDD.

To the best of our knowledge, this is the first fMRI study specifically addressing aberrancy in brain activation in males with MDD, and sex differences in cognitive control in adolescents with MDD. It was hypothesized that there would be a significant sex difference in brain activation associated with cognitive control in the affected group. Despite using an uncorrected statistical threshold, a previous fMRI study located the sex differences in cognitive control responding to negative stimuli to the supramarginal gyrus, amygdala, and orbitofrontal cortex (13). Interestingly, these regions are also among the core areas of pathology of MDD according to Mayberg’s limbic-cortical dysregulation model (39). Sex differences in cognitive control in depressed patients would help to explain the difference in the manifestation of MDD in males and females. Specifically, we hypothesized that group-by-sex interactions in brain activation subserving emotional cognitive control would be found in the aforementioned brain regions: the supramarginal gyrus, amygdala, and orbitofrontal cortex. However, given the lack of previous studies, we conducted whole-brain analysis to identify patterns of task-induced activity and then tested for group-by-sex interactions in those regions. Initially, all participants were investigated, followed by an analysis in male participants alone. Results from the female participants alone have been published elsewhere (38).

## Materials and Methods

### Participants

Patients with a diagnosis of MDD were recruited from the Improving Mood with Psychoanalytic and Cognitive Therapies (IMPACT), a pragmatic effectiveness clinical trial (40) in East Anglia, North London, and North West England, with patients randomized to one of three different treatment arms to determine the short-to-medium term efficacy of conversational therapies. A subsample of patients from the East Anglia region were invited to participate in an adjunctive study, MR-IMPACT, aimed at exploring the neural mechanisms underlying MDD and recovery following treatment using MRI (41). Healthy control participants matched for age, sex, and handedness were also recruited in the MR-IMPACT study (41). Randomization was first performed in patients, then MRI neural images were taken, which were followed by conversational therapies.

General inclusion criteria for patients in the IMPACT trial (40) and MR-IMPACT study (41) were: aged 11 through 17 years; diagnosis of current moderate-to-severe Diagnostic and Statistical Manual of Mental Disorders (DSM-IV) MDD, as determined by patient and parent interviews with the Kiddie Schedule for Affective Disorders and Schizophrenia-Present and Lifetime version; and score of 27 or above on the self-reported Moods and Feelings Questionnaire (42). Exclusion criteria for the IMPACT trial (40) included: generalized learning difficulties; pervasive developmental disorder; bipolar I disorder; schizophrenia; eating disorder; pregnancy; and currently taking a medication that may interact with a selective serotonin reuptake inhibitor. Additional exclusion criteria of the MR-IMPACT study for all participants (i.e., including controls) were as follows: alcohol dependence; drug dependence; MRI contraindication; brain abnormality; and intolerance to the MRI environment (41).

Overall, 126 patients and 40 controls participated in the study. Ninety-four female patients and 29 female controls were enrolled, although some participants were subsequently excluded from the analysis for the following reasons: brain abnormality (1 patient), dental braces (7 patients and 2 controls), schizophrenia comorbidity (1 patient), excessive (>3 SDs) motion during fMRI scanning (1 patient and 2 controls), and poor (>3 SDs) behavioral performance on the AGNG task (2 patients and 1 control). Thirty-two male patients and 11 male controls were enrolled. Similarly, some male participants were excluded for the following reasons: brain abnormality (one patient and one control), dental braces (three patients), schizophrenia comorbidity (two patients), excessive motion during scanning (one patient), and poor behavioral performance on the AGNG task (one patient). Overall, 82 female patients and 24 female controls along with 24 male patients and 10 male controls were included in the analysis. The study was conducted in accordance with the Declaration of Helsinki (https://www.wma.net/what-we-do/medical-ethics/declaration-of-helsinki/). Ethical approval was given by the Cambridgeshire 2 Research Ethics Committee, UK (REC reference: 09/H0308/168).

As the study recruited participants through a pragmatic effectiveness trial, more women than men were enrolled in this study resulting in an unbalanced design with restricted power for addressing sex effects. Likewise, the number of male relative to female controls enrolled was fewer.

### Affective Go/No-Go Task

**Figure 1:**
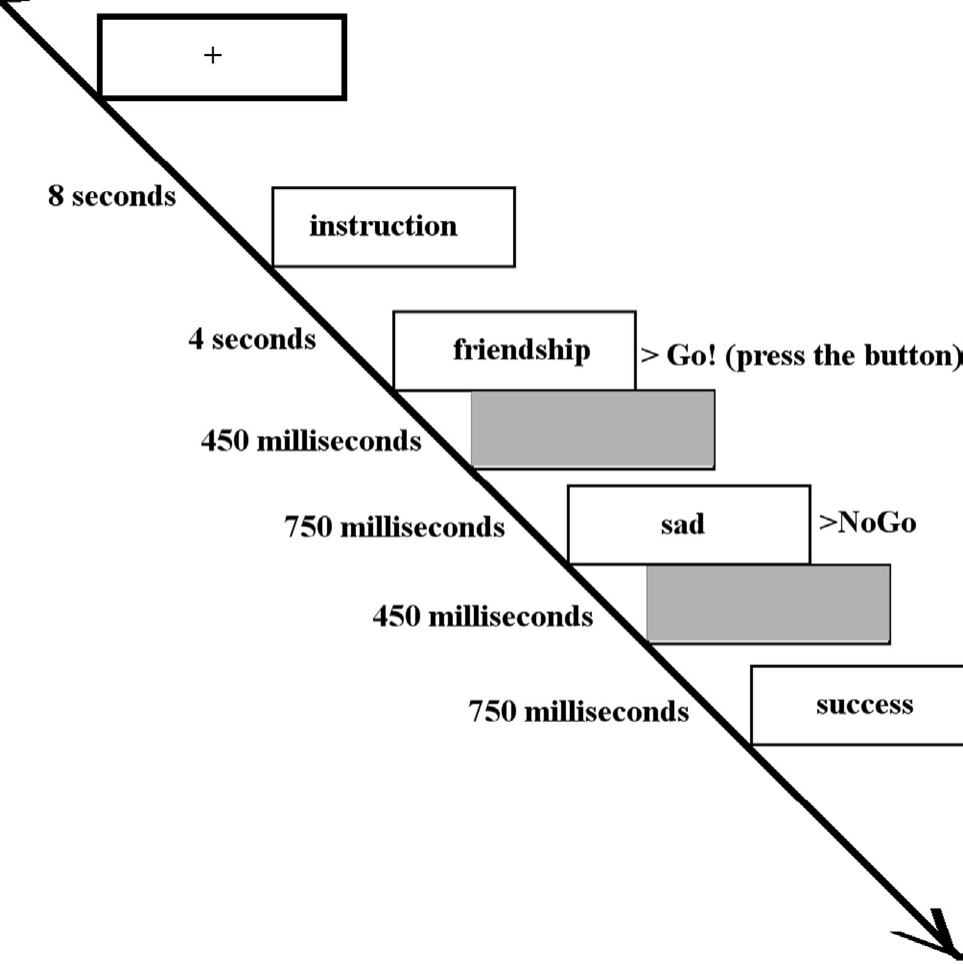
The inter-block interval was 12 s with the first 4 s of each block used to present the instructions (in this case, “Press for happy words. Ignore sad words.”). Each word was presented for 450 ms with a 750 ms interstimulus interval. Participants were asked to press a button when presented with a target word (in this case, happy words) and inhibit responses to distractor words (in this case, sad words).

Happy, sad, and neutral words were presented to participants during fMRI data acquisition using a block paradigm task design (37) (Figure 1). Words did not differ in terms of length or usage frequency (43). There were seven types of blocks each repeating three times, consisting of randomly ordered targets/distractors (10 of each type): sad/neutral (SN), sad/happy (SH), neutral/sad, neutral/happy, happy/sad (HS), happy/neutral (HN), and neutral italic/plain. Equal numbers of happy and sad words were used with mood induction unlikely to occur. As a result, neural responses of the participants were likely to be related to cognitive control instead of affective response. The inter-block interval was 12 s with the first 4 s of each block used to present the instructions for that block. Each word was presented for 450 ms with a 750 ms interstimulus interval (41). Participants were asked to press a button with their right index finger when presented with a target word (“go”) and to inhibit responses to distractor words (“no-go”). All participants completed a go/no-go practice task (living versus non-living stimuli) prior to scanning. The following analyses were based on two contrasts: a *happy distractor contrast* (SH–SN) and a *sad distractor contrast* (HS–HN) with targets fixed and distractors differing in valence.

### Behavioral Data Analysis

Behavioral output tailored to the *happy distractor contrast* and the *sad distractor contrast* was recorded to capture three variables: mean response time of correct go, incorrect go (omission error), and incorrect no-go (commission error). For instance, mean response time of the *sad distractor contrast* was defined as the mean reaction time of the HS condition minus the mean reaction time of the HN condition. Likewise, omission error of the *happy distractor contrast* was defined as the number of the omission errors in the SH condition minus the number of the omission errors in the SN condition. The timing of responses was measured when participants released the button. Analysis of covariance was performed in SPSS (Statistical Package for Social Science, version 21) on the behavioral measures, with group (depressed/healthy) and sex as independent variables, and age as a covariate. Group, sex, and age effects, as well as group-by-sex and group-by-age interactions were then explored in a single model with a statistical threshold for significance of *p* < 0.05.

### fMRI Analysis

#### fMRI Data Acquisition

Participants were scanned on a 3-T Magnetom Trio Tim MRI scanner (Siemens, Surrey, England) at the Wolfson Brain Imaging Centre, University of Cambridge, UK. Thirty-two slices of data parallel to the anterior–posterior commissure comprised each three-dimensional volume acquired continuously during the task. Acquisition parameters were: echo time, TE = 30 ms; repetition time, TR = 2 s; flip angle = 78°; field of view = 192 mm × 120 mm; and 3.0 mm × 3.0 mm × 3.0 mm voxel size with an interleaved slice acquisition (41).

##### Within-Subject fMRI Data Preprocessing

Imaging data were preprocessed with FEAT (FMRI Expert Analysis Tool, version 6.00) in FSL 5.0.6 (FMRIB’s Software Library, www.fmrib.ox.ac.uk/fsl). Participant head motion was corrected with MCFLIRT (Motion Correction FMRIB’s Linear Image Registration Tool) (44), interleaved slice timing was corrected with Fourier-space time-series phase-shifting, non-brain tissue was removed by Brain Extraction Tool (45), spatial smoothing was performed with a Gaussian kernel of FWHM 6 mm, grand-mean intensity of data was normalized by a single multiplicative factor, and high-pass temporal filtering with 90 s cutoff was performed.

##### Between-Subject fMRI Data Analysis

Following initial within-subject (first level) analysis to generate *z*-statistic maps, all maps were normalized to the standard Montreal Neurological Institute (MNI) space (46) by FLIRT (FMRIB’s Linear Image Registration Tool) (44, 47).

To accommodate the possibility of unequal variances between males and females due to the unbalanced design, we used the Aspin–Welch statistic and permutation inference, as implemented in the tool Permutation Analysis of Linear Models (PALM), part of the FSL package (https://fsl.fmrib.ox.ac.uk/fsl/fslwiki/PALM) (48).

With all participants, a whole-brain Aspin–Welch unequal variance test controlling for age determined significant mean activation and deactivation patterns of the two contrasts such that for the *happy (or sad) distractor contrast*, the activation pattern referred to the brain regions more activated to the SH (or HS) condition than the SN (or HN) condition, and the deactivation pattern referred to the brain regions more activated to the SN (or HN) condition than the SH (or HS) condition. Subsequent between-subject analysis was restricted to these mean activated or deactivated regions. The Aspin–Welch unequal variance test was then performed with group (depressed/healthy) and sex as independent variables, and age as a covariate. Group, sex, and age effects as well as group-by-sex and group-by-age interactions were examined. Significant statistical results were identified using threshold-free cluster enhancement at a threshold of *p* < 0.05, family-wise error rate corrected.

To explore the aberrancy of activation within male participants, data were subsequently analyzed solely in male participants. Since patients and controls did not differ much in sample size, Aspin–Welch unequal variance test was not used in this analysis. Instead, FEAT with FLAME (FMRIB’s Local Analysis of Mixed Effects) was used in a whole-brain one-sample *t*-test controlling for age, to determine significant patterns of mean activation and deactivation. Then, restricting the analysis to these mean activated or deactivated regions, group effects controlling for age were explored. Again, significant statistical results were obtained using TFCE at a threshold of *p* < 0.05, family-wise error rate corrected.

### Psychophysiological Interactions (PPIs)

Psychophysiological interaction analysis identifies regions that increase their connectivity with a seed region of interest during task performance (49). That is, using the fMRI data with a seed region specified, PPI detects brain areas whose activity depends on an interaction between a psychological factor (task performance) and physiological factor (time course of the seed region) (49).

For consistency with univariate approaches, regions showing significant group effects in between-subject fMRI analyses were used as seeds. First, this seed region was transformed into the individual fMRI acquisition space of each participant using FNIRT (FMRIB’s non-linear image registration tool). It is likely that the significant group effect might reside in a relative small region compared to the whole-brain. The precision of this transformation in such a small region is critical to the following analyses, and thus non-linear (FNIRT) instead of linear (FLIRT) transformation was used to ensure sufficient deformation (http://fsl.fmrib.ox.ac.uk/fsl/fslwiki/FNIRT) at the cost of computation time. The time course of the transformed region was extracted from each individual dataset.

Second, within-subject analysis was carried out with the time course extracted from the seed as a factor denoted “physiological regressor.” The *happy distractor contrast* and the *sad distractor contrast* denoted the “psychological regressors.” The interaction between the physiological and psychological regressors was then defined as the PPI. Finally, a between-subject FEAT analysis was performed with group effects, age effects, and the group-by-age interactions examined. Significant statistical results were obtained using TFCE at a threshold of *p* < 0.05, family-wise error rate corrected.

## Results

### Demographic and Behavioral Results

**Figure 2:**
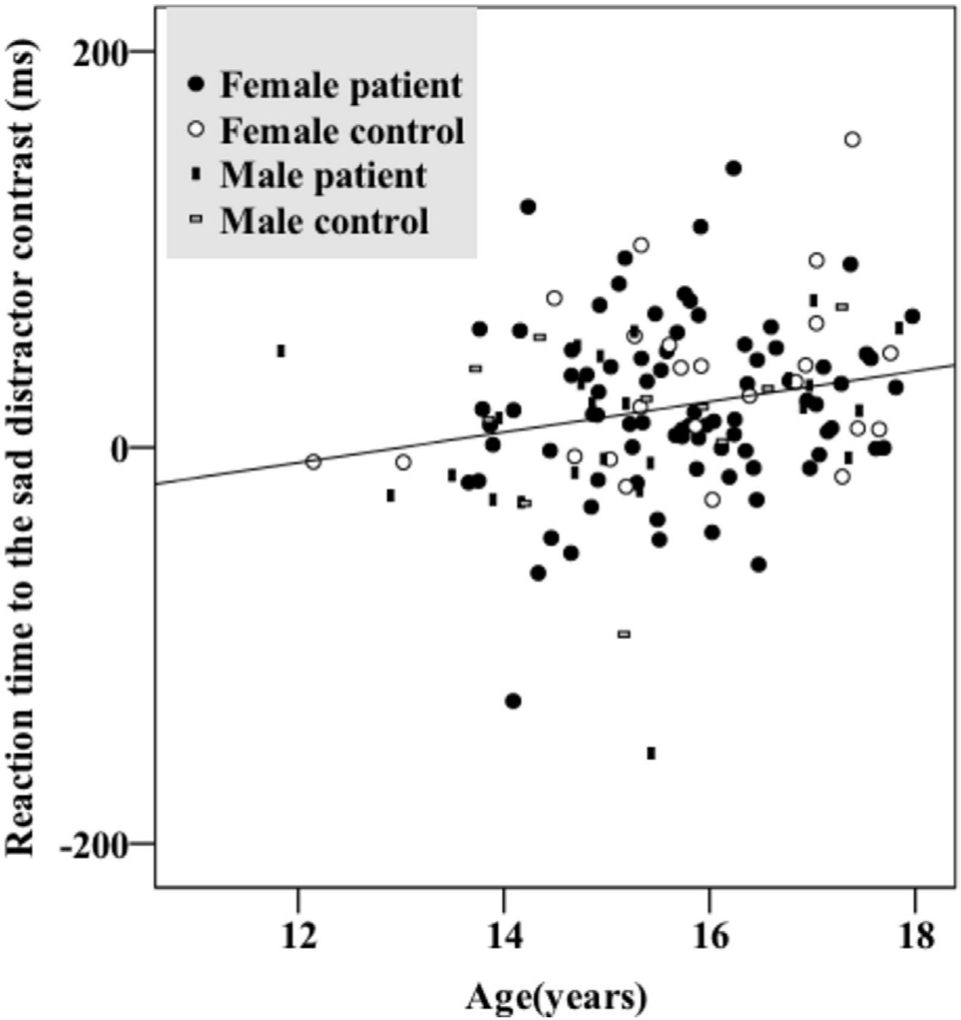
Older participants had longer reaction times responding to the *sad distractor contrast*. There were no significant group (depressed/healthy) or sex effects. Female patients, female controls, male patients, and male controls all showed similar patterns.

There were no significant sex differences in demographic and behavioral characteristics (age, intelligence, handedness, trait anxiety, MDD severity, number of commission errors, number of omission errors, reaction time) with the exception that female adolescents had higher levels of state anxiety than male adolescents (Table 1). There were no significant sex differences (*t*/df/*p* = 1.50/34/0.142) in antidepressant usage (mean fluoxetine equivalent dose × duration): 19.52 mg × 2.43 months in girls and 18.41 mg × 1.34 months in boys. There was a significant age effect [*F*(1, 134) = 5.250, *p* = 0.024] with older participants taking a longer time to respond to the happy targets (in the *sad distractor contrast*, i.e., during the sad versus neutral distractors) (Table 2; Figure 2).

**Table 1:** Between-sex differences in demographic and baseline characteristics.

	Depressed patients			Healthy controls		
	Females, mean/SD	Males, mean/SD	Between-sex difference, *t*/df/*p*	Females, mean/SD	Males, mean/SD	Between-sex difference, *t*/df/*p*
Number	82	24	–	24	10	–
Age (years)	15.72/1.10	15.25/1.52	1.68/104/0.10	15.89/1.42	15.26/1.22	1.23/32/0.23
Estimated WASI IQ	97.83/12.02	98.25/9.89	−0.14/31.59/0.89	100.79/10.85	99.60/7.96	0.31/32/0.76
Edinburgh Handedness Inventory	58.66/54.11	67.92/52.50	−0.74/104/0.46	62.46/57.86	60.00/58.88	0.11/32/0.91
STAI-State	47.70/10.49	39.79/10.05	3.28/104/0.001***	28.92/6.43	27.50/5.64	0.61/32/0.55
STAI-Trait	61.44/7.41	57.17/10.12	1.92/30.57/0.06	31.13/6.78	29.90/6.24	0.49/32/0.63
SMFQ	18.11/5.02	16.38/5.20	1.48/104/0.14	2.63/2.02	3.30/2.00	−0.89/32/0.38
Happy distractor: commission	1.67/3.01	1.00/4.74	0.66/28.63/0.52	1.50/2.54	0.30/2.45	1.27/32/0.21
Happy distractor: omission	−0.48/2.86	−0.04/3.56	−0.62/104/0.54	−1.00/2.36	0.10/0.99	−1.91/31.95/0.07
Happy distractor: reaction time	−12.63/52.28	3.97/46.63	−1.40/104/0.16	−7.64/42.14	−6.71/66.22	−0.05/32/0.96
Sad distractor: commission	0.91/3.21	1.21/3.54	−0.39/104/0.70	0.67/4.47	1.40/2.55	−0.48/32/0.63
Sad distractor: omission	0.37/3.54	0.71/3.07	−0.43/104/0.67	0.33/3.19	−0.60/2.95	0.79/32/0.43
Sad distractor: reaction time	20.57/43.66	9.81/46.35	1.05/104/0.30	32.99/43.81	13.52/46.75	1.16/32/0.26
Commission error—correct targets	−138.32/35.39	−131.79/41.49	−0.76/104/0.45	−152.17/20.80	−140.20/24.68	−1.45/32/0.16

*** p ≤ 0.001; Bold font are significant results.

**Table 2:** Behavioral results.

Effect	Commission (*F*/*p*)	Omission (*F*/*p*)	Reaction time (*F*/*p*)
**(A). The *happy distractor contrast***			
Group (depressed/healthy)	1.342/0.249	3.563/0.061	0.574/0.450
Sex	1.411/0.237	1.801/0.182	0.253/0.616
Age	0.065/0.800	0.195/0.659	1.621/0.205
Group × sex	0.021/0.886	0.785/0.377	0.745/0.390
Group × age	1.229/0.270	3.490/0.064	0.612/0.436
**(B) The *sad distractor contrast***			
Group (depressed/healthy)	0.270/0.604	0.116/0.734	0.152/0.697
Sex	0.805/0.371	0.186/0.667	1.184/0.279
Age	1.495/0.224	0.032/0.858	5.250/0.024*
Group × sex	0.168/0.682	0.843/0.360	0.084/0.772
Group × age	0.266/0.607	0.180/0.672	0.214/0.644

* p ≤ 0.05; Bold font are significant results.

### Results from the Analysis of All Participants

**Figure 3:**
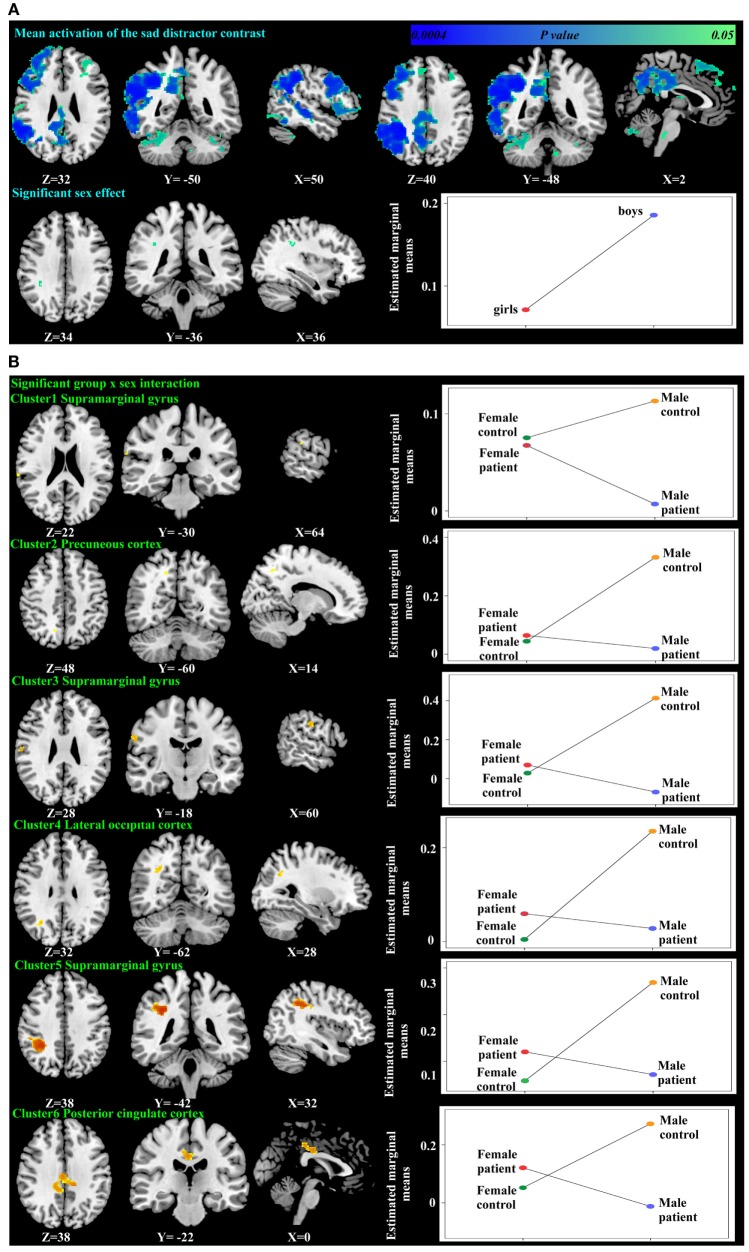
Significant mean activation, sex effect **(A)** and group-by-sex interaction **(B)** responding to the *sad distractor contrast* in all participants. Box plots depicted estimated marginal means of the percent signal changes extracted from the regions showing significant sex effect in the supramarginal gyrus **(A)**, and a group-by-sex interaction in the supramarginal gyrus, precuneus cortex, lateral occipital cortex, and the posterior cingulate cortex **(B)**.

No significant within-group mean activations or deactivations were found in response to the *happy distractor contrast* suggesting similar brain activation patterns occurring for happy compared to neutral distractors. In response to the *sad distractor contrast*, significant within-group mean activation occurred in regions of posterior cingulate, supramarginal gyrus and dorsolateral prefrontal cortex, similar to the well-acknowledged frontoparietal activated region in the go/no-go task (50). No significant mean deactivation regions were identified (Table 3; Figure 3).

**Table 3:** Significant mean activation, sex effect and group × sex interactions responding to the *sad distractor contrast* in all participants.

Cluster	Cluster size (voxels)	Maximum *z* value	Peak Montreal Neurological Institute coordinates (*X*, *Y*, *Z* in mm)	Location of the peak
Mean activation	10,237	2.82	(36, **−**40, 34)	Right supramarginal gyrus
	7,804	2.5	(30, 6, 44)	Right DLPFC
	1,761	2.06	(**−**22, **−**74, **−**28)	Left cerebellum
	668	1.8	(12, **−**8, 16)	Right thalamus
	268	1.81	(**−**36, 32, 34)	Left DLPFC
	198	1.75	(**−**6, **−**12, **−**6)	Left thalamus
	64	1.67	(2, **−**32, **−**38)	Brain stem
	27	1.67	(8, **−**28, **−**10)	Brain stem
	18	1.7	(**−**10, **−**46, **−**44)	Brain stem
Sex effect	18	1.72	(36, −36, 34)	Right supramarginal gyrus
Group × sex				
Cluster 1	5	1.65	(64, −30, 22)	Right supramarginal gyrus
Cluster 2	10	1.66	(14, −60, 48)	Right precuneus cortex
Cluster 3	32	1.84	(60, −18, 28)	Right supramarginal gyrus
Cluster 4	43	1.8	(28, −62, 32)	Right lateral occipital cortex
Cluster 5	429	2.37	(32, −42, 38)	Right supramarginal gyrus
Cluster 6	429	2	(−12, −34, 38)	Left posterior cingulate cortex

Using PALM and restricting the analysis to the mean activation regions described above did not reveal any significant group, age effects, or group-by-age interactions. Consequently, despite the longer reaction times found in the older participants, their brain activation did not differ significantly from the younger participants. A significant sex effect was found in the supramarginal gyrus, and significant group-by-sex interactions were found in the supramarginal gyrus and posterior cingulate (Table 3; Figure 3). Mean percent signal changes were then extracted from regions with significant results. *Post hoc* inspection of the extracted data revealed that the reductions in activation in males with MDD relative to healthy male adolescents were driving the majority of the significant group × sex interaction results (Figure 3; Table 4). Although there was a significant sex difference in STAI-S state anxiety (Table 1), no significant correlations between STAI-S and these percent signal changes were found.

**Table 4:** *Post hoc* tests of the percent signal changes from the regions showing significant group × sex interactions.

Cluster	*Post hoc* *t* value in girls (comparing patients with controls)	*p*-Value	*Post hoc* *t* value in boys (comparing patients with controls)	*p*-Value
		df = 103		df = 31
Group × sex				
Cluster 1	0.048	1.000	−1.101	0.556
Cluster 2	0.841	0.800	−3.649	0.002**
Cluster 3	0.697	0.968	−5.133	0.000032****
Cluster 4	1.585	0.230	−2.987	0.01**
Cluster 5	1.636	0.206	−3.014	0.01**
Cluster 6	1.750	0.172	−3.158	0.008**

** p ≤ 0.01; **** p ≤ 0.0001; Bold font are significant results.

### Brain Activation in Male Participants

**Figure 4:**
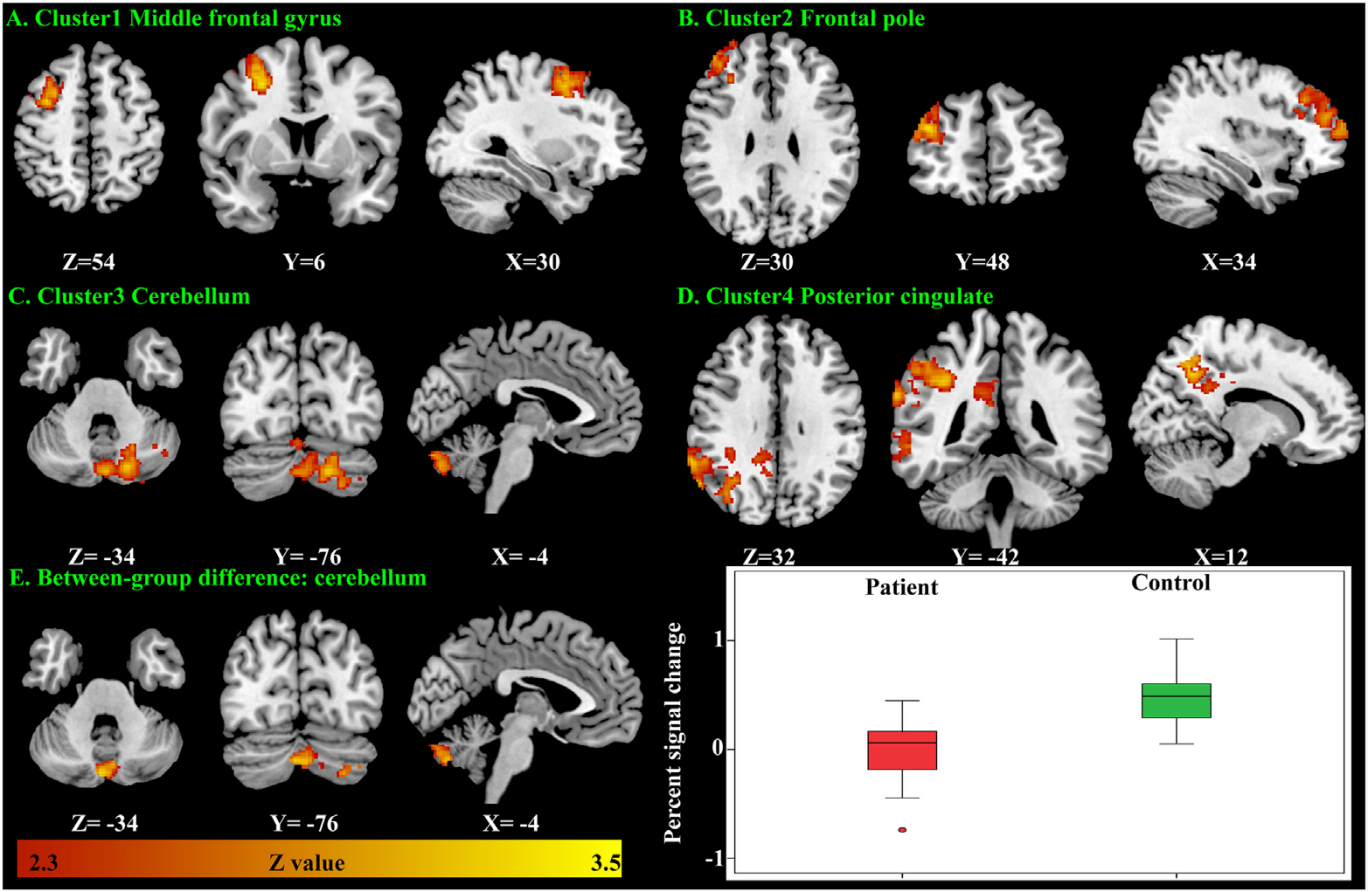
Significant brain activation responding to the *sad distractor contrast* in the male adolescents. Significant mean activation **(A–D)** and significant case–control difference of these mean activation regions **(E)**. A box plot showed higher brain activation (extracted percent signal change) in the male controls’ cerebellum when compared with the male patients.

There were no significant within-group mean activation or deactivations for the *happy distractor contrast*. However, significant mean activations were found in the frontal pole, middle frontal gyrus, posterior cingulate cortex (PCC), and the cerebellum responding to the *sad distractor contrast*. Within these mean activation regions, male adolescents with MDD were found to exhibit lower brain activations than healthy male adolescents in the Crus I and Crus II regions of the cerebellum (Table 5; Figure 4).

**Table 5:** Significant fMRI mean activation, group difference, and group × age interaction of the connectivity between the cerebellum and the superior frontal gyrus responding to the *sad distractor contrast*.

Cluster	Cluster size (voxels)	Maximum *z* value	Peak Montreal Neurological Institute coordinates (*X*, *Y*, *Z* in mm)	Location of the peak
Male mean activation				
Cluster1	662	3.45	(26, 4, 46)	Right middle frontal gyrus
Cluster 2	1,130	3.35	(38, 48, 24)	Right frontal pole
Cluster 3	1,254	3.46	(−18, −70. −44)	Cerebellum
Cluster 4	3,540	3.41	(58, −50, 24)	Right angular gyrus
Male case–control difference of the activation map (patient < control)	395	3.47	(4, −80, −36)	Cerebellum
Group × age interaction of the psychophysiological interaction connectivity	560	3.21	(38, −6, 48)	Right precentral gyrus

### PPI Analysis of Male Participants

**Figure 5:**
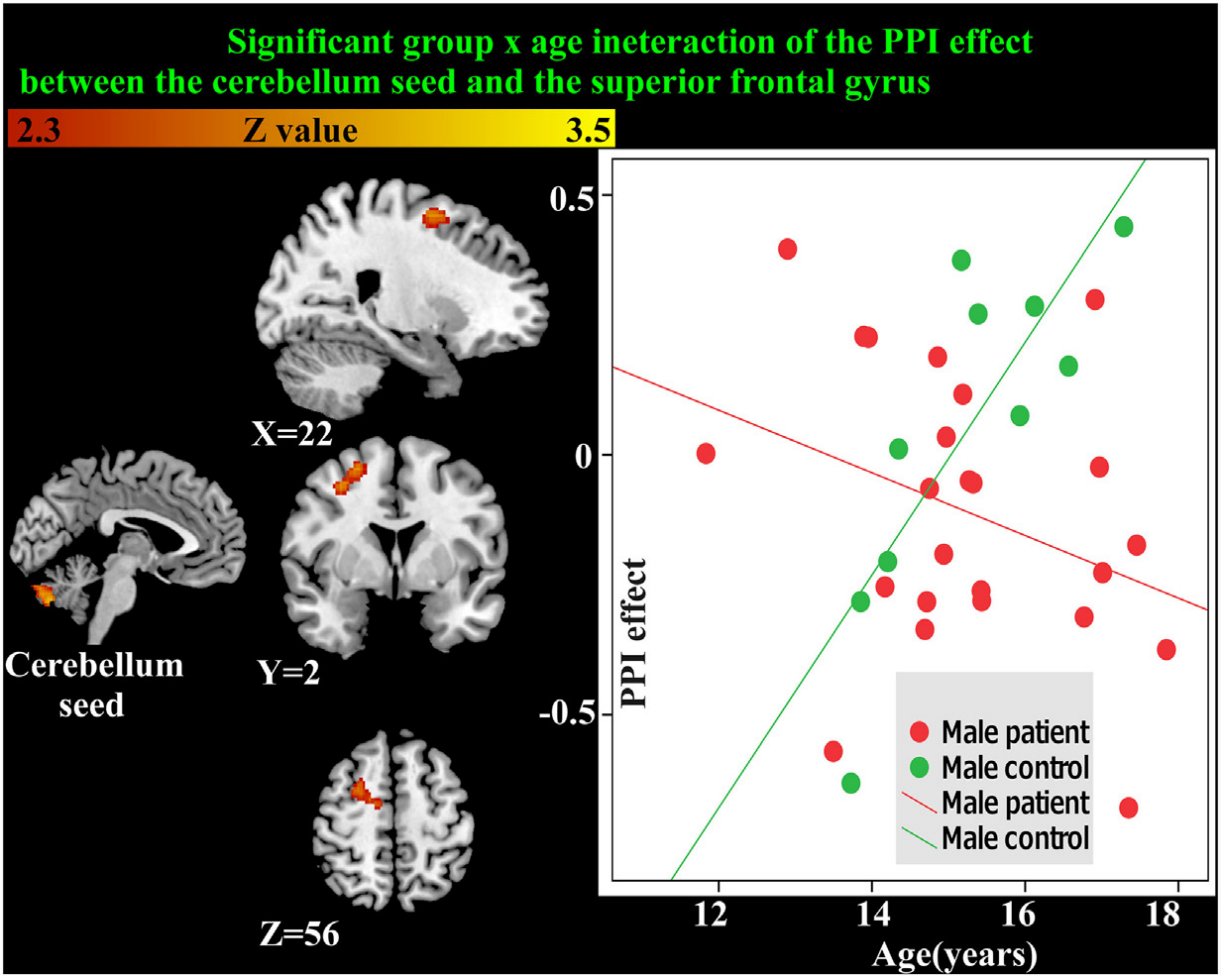
Significant group × age interaction of the psychophysiological interaction (PPI) connectivity between the cerebellum seed and superior frontal gyrus is shown. With age, connectivity increases in strength in male controls but decreases in strength in males with major depressive disorder.

As the cerebellum was found to show a significant group difference, this region was chosen as a seed region for the PPI analysis. No significant group or age effects were found. However, there was a significant group-by-age interaction between the cerebellum seed and the superior frontal gyrus (Table 5; Figure 5). The PPI effect showed that compared with younger participants, connectivity between the cerebellum and superior frontal gyrus increased in strength across age in male controls, but decreased in strength with age in males with MDD (Figure 5). Taken together, differential functional responses associated with MDD and age-related changes of brain connectivity were found in the cerebellum of male adolescents.

All of the significant results identified in the primary analysis were checked for medication effects by *t*-tests between the group of patients using antidepressants (*n* = 36) and the group of patients not using antidepressants (*n* = 70). *t*-Statistics, *p* values, means, and SDs associated with these tests are shown in the Table S1 in Supplementary Material. Patients with and without antidepressant usage did not differ significantly in terms of behavior or brain activation.

## Discussion

In response to the *sad distractor contrast*, significant sex differences (in the supramarginal gyrus), group-by-sex interactions (in the supramarginal gyrus and the PCC) and group differences in males (cerebellum) were found. Furthermore, a significant group-by-age interaction in connectivity between the cerebellum and superior frontal gyrus was observed.

### Response to the Happy Targets Intermixed with the Sad versus Neutral Distractors

Results from a previous study using an affective go/no-go task suggests that there is no significant developmental change of the reaction time difference between the negative image distractors and the positive or neutral images in the age range of 11–25 years (51). However, another go/no-go overlap task reports significant reaction time differences between fearful face distractors and happy or neutral face distractors in younger adolescents (11- to 12-year olds) which diminished in older adolescents (17- to 18-year olds) (52). Our data instead associated increased age with greater response time differences toward the sad versus neutral word distractors (in the *sad distractor contrast*, HS–HN) (Table 2; Figure 2). This inconsistency could be related to the fact that neutral targets were used in the previous two studies whereas happy targets were used in our work. Furthermore, all three studies used cross-sectional designs. Future research is warranted before these results can be comprehensively interpreted.

### Interaction Effect Regions: The Supramarginal Gyrus

The supramarginal gyrus is located within the inferior parietal lobe, a region thought to be involved in attention, processing of written language, and working memory of emotional stimuli (53). Several studies suggest a relationship between MDD and supramarginal gyrus (54, 55). A meta-analysis indicates aberrant supramarginal gyrus activation toward positive emotional stimuli associated with MDD (54). Furthermore, aberrant connectivity has been shown between the medial prefrontal cortex and the supramarginal gyrus in adolescents with MDD (55).

Compared with women, men have larger total volume of the inferior parietal lobule (supramarginal gyrus and angular gyrus) (56). There is also evidence of sex differences in supramarginal gyrus function. Higher regional homogeneity (ReHo) of the fMRI time series in the supramarginal gyrus has been reported in female compared to male adults (57). In the supramarginal gyrus, men also show higher activation in response to negative words and lower activation in response to positive words as compared with women (58). Moreover, compared to women, hyperactivation of the supramarginal gyrus has been found during the cognitive control of negative stimuli in men (13). Our finding of a sex effect and group-by-sex interaction in the supramarginal gyrus extends these previous results and highlights the supra marginal gurus as a potentially key region contributing to sex differences in MDD.

### Interaction Effect Regions: The PCC

The PCC is involved in risky decision-making (59), daydreaming, autobiographical memory, attention, and conscious awareness (60). This study showed a significant group-by-sex interaction in PCC. Previous literature has associated the PCC with MDD and sex effects; an overactive PCC has been observed in treatment-resistant adult MDD (60), and a meta-analysis also demonstrates that men have larger gray matter volume in the PCC as compared to women (61). However, to the best of our knowledge, no previous study has addressed the group-by-sex interaction in PCC.

### Cerebellar Dysfunction in Males with MDD

Although initially considered as a motor coordinator, the cerebellum is now known to play a role in emotional regulation and cognition, supported by its widespread connections to the limbic system, the frontal, parietal, prefrontal, occipital, and temporal cortex. The cerebellum is activated in a variety of mental activities, including facial recognition, emotion attribution, theory of mind attributions, directed attention, memory, and empathy (62, 63) and has been referred to as the “emotional pacemaker” (64). Indeed, the cerebellar cognitive affective syndrome following posterior cerebellar lobe lesion includes negative symptoms such as passivity, blunted affect, and withdrawal. In an early PET study, Dolan et al. reported increased regional cerebral blood flow of the cerebellar vermis in depressed adult patients with cognitive disturbance (65). Furthermore, in an fMRI meta-analysis, the cerebellum has been identified as an important area of dysfunction in MDD (66). Other meta-analyses suggest decreased ReHo in the left cerebellum (67) and aberrant cerebellar activation toward positive and negative emotional stimuli in MDD (54).

It has been proposed that the relatively protracted developmental course of the cerebellum may be associated with its vulnerability to the effects of mental disorders (68). A developmental difference exists between sexes with the total cerebellum volume peaks at age 15.6 years in boys in contrast to 11.8 years in girls (68). A prior study showed hyperactivation of the cerebellum in response to the positive stimuli in depressed compared to healthy adolescents (69). Another study demonstrated aberrant activation of the cerebellum responding to the negative distractor in adolescents with family history of MDD when compared to healthy controls (70). Similarly, we have identified hypoactive cerebellum responding to the *sad distractor contrast* in male adolescents with MDD.

We demonstrated a significant group-by-age interaction of the connectivity between cerebellum Crus I, Crus II, and the superior frontal gyrus in male adolescents. Superior frontal gyrus along with Crus I and Crus II of the cerebellum (71) is part of the default network. Indeed, a meta-analysis supports the importance of the aberrant resting-state connectivity between the cerebellum and other default network regions responsible for internally oriented and self-referential thinking in MDD (mean age 37.77 years old) (72). Examples include: higher resting-state connectivity between cerebellum and middle temporal gyrus (73, 74), and between the cerebellum and superior temporal pole (75). A multivariate pattern analysis also suggested that the resting-state connection of the cerebellum is among the most discriminating regions separating healthy from depressed adults (75). To the best of our knowledge, there is no longitudinal study addressing the interaction between the connectivity of the cerebellum and development. However, similar to adult MDD, fronto-cerebellar dysregulation has been hypothesized to be a pathophysiological mechanism of adolescent MDD (69). Aberrant resting-state connectivity between the cerebellum and other default network regions has also been found in geriatric MDD (76). The developmental trajectory of the connections in the cerebellum awaits further exploration.

### Limitations

Reflecting the skewed prevalence of MDD, the sample size of the male patient group was relatively limited. Furthermore, the number of males in the control group was also limited. Presumably, the restricted number of males might be related to the failure to find significant group-by-sex interactions in the amygdala and orbitofrontal cortex as hypothesized. Future studies examining sex differences in adolescent MDD with larger male control groups are needed. Since our significant results were not all hypothesized (e.g., cerebellum), they should be considered preliminary until replicated. Furthermore, although alcohol and drug dependence were among the exclusion criteria, we were unable to explore the possible confounding effects of alcohol and drug since details of frequency or severity of usage were not recorded.

Most of the significant findings in this study reside in the neuroimaging data, but not in the behavioral data. Our task was designed to be primarily sensitive to brain activation rather than to measure variability in behavior. Discrepancy between behavioral performance and brain activations is frequently reported in the psychiatric fMRI literature, possibly related to the higher sensitivity of brain signals to detect case–control differences or alternative neural strategies to achieve comparable performances in the patient and control groups (38).

Finally, a cross-sectional design was used in this research and although we examine age effects and interactions, we cannot describe the developmental trajectory of our findings with the same confidence that could be drawn from a longitudinal study. Furthermore, we cannot exclude the potential effect of normal developmental sex differences in the cerebellum. Also, pubertal stage was not considered in the analysis. Future research should incorporate these factors into a longitudinal design.

### Future Research: The Default Network

Although network inference was restricted by task design and the fact that the majority of the analyses were univariate in nature, the supramarginal gyrus, PCC, Crus I, and Crus II of the cerebellum, and superior frontal gyrus all contribute to the default network. Indeed, aberrant brain activation of the default network toward negative pictures has been found with an increase of self-referential focus in MDD (77). Current research also indirectly indicates the importance of default network in the sex differences in MDD. First, men are found to be more liable to persistent MDD whereas women are more likely to suffer from episodic MDD (8). Furthermore, it has been proposed that the default network acts as an overarching mechanism for the impaired cognitive control of recurrent MDD (78). Second, the default network has been regarded as the key to cognitive decline in MDD (79) with men more liable to the cognitive effects of the disorder leading to dementia (80, 81). Future research might consider using multivariate analysis to directly explore the relationship between the default network and sex differences in MDD.

## Conclusion

Our study provides the first neuroimaging evidence of sex differences observed with functional imaging in adolescent MDD. In response to sad relative to neutral distractors, group-by-sex interactions were found in the supramarginal gyrus and the PCC. In particular, novel aberrant brain activation in the cerebellum with a group-by-age interaction of its connectivity with the superior frontal gyrus was identified in male adolescents with MDD. Future research may explore the neural mechanisms of sex differences and their relationships with clinical symptomology.

## Ethics Statement

This study was carried out in accordance with the recommendations of Cambridgeshire 2 Research Ethics Committee with written informed consent from all subjects. All subjects gave written informed consent in accordance with the Declaration of Helsinki. The protocol was approved by the Cambridgeshire 2 Research Ethics Committee.

## Author Contributions

EB, BL, BS, IG, and JS conceived and designed the experiment. CH, JG, RH, and AN collected the data. J-YC, CO, and RT analyzed the data, with advice from JS and GM. J-YC, JS, RE, and GM drafted the manuscript. All the authors critically reviewed the manuscript.

## Conflict of Interest Statement

BS reports personal fees from Cambridge Cognition, personal fees and other from Lundbeck, personal fees from Servier, grants from J&J Janssen, other from Otsuka, personal fees from Peak (Brainbow), outside the submitted work; EB works half-time for the University of Cambridge and half-time for GlaxoSmithKline. He holds stock in GlaxoSmithKline; IG reports personal fees from Lundbeck and holds grants from the Wellcome Trust and the Friends of Peterhouse Charity and NIHR-HTA. All other authors declare no conflict of interest.
