# Supplementary material for: Adolescent Major Depressive Disorder: Neuroimaging Evidence of Sex Difference during an Affective Go/No-Go Task
Source: Front Psychiatry. 2017 Jul 11;8:119. doi: 10.3389/fpsyt.2017.00119 (PMC5504124; doi:10.3389/fpsyt.2017.00119)
Supplement: Supplementary file 1 [file Table_1.DOCX]

|  | Patients using antidepressant  mean/sd | Patients without antidepressant  mean/sd | Between-group difference  *t*/df */p* |
| --- | --- | --- | --- |
| Reaction time to the sad distractor contrast (ms) | 22.03/31.36 | 16.13/49.74 | 0.65/104/0.52 |
| Significant sex effect responding to the sad distractor contrast (percent signal change) | 0.08/0.23 | 0.09/0.22 | -0.31/104/0.76 |
| Significant group-by-sex effect responding to the sad distractor contrast Cluster1 (percent signal change) | -0.002/0.33 | 0.08/0.38 | -1.13/104/0.26 |
| Significant group-by-sex effect responding to the sad distractor contrast Cluster2 (percent signal change) | 0.05/0.20 | 0.05/0.20 | -0.02/104/0.98 |
| Significant group-by-sex effect responding to the sad distractor contrast Cluster3 (percent signal change) | 0.08/0.34 | 0.01/0.29 | 1.11/104/0.27 |
| Significant group-by-sex effect responding to the sad distractor contrast Cluster4 (percent signal change) | 0.06/0.20 | 0.05/0.18 | 0.13/104/0.90 |
| Significant group-by-sex effect responding to the sad distractor contrast Cluster5 (percent signal change) | 0.11/0.22 | 0.11/0.18 | 0.07/104/0.95 |
| Significant group-by-sex effect responding to the sad distractor contrast Cluster6 (percent signal change) | 0.05/0.20 | 0.11/0.22 | -1.34/104/0.18 |
| Significant group effect responding to the sad distractor contrast in the male adolescents (percent signal change) | -0.07/0.24 | 0.04/0.29 | -0.98/22/0.34 |
| Significant group-by-age effect responding to the sad distractor contrast in the male adolescents (PPI connectivity) | -0.09/0.28 | -0.13/0.27 | 0.34/22/0.74 |
